# Editorial: Proteases of the prokaryotic cell envelope

**DOI:** 10.3389/fmicb.2022.988067

**Published:** 2022-08-02

**Authors:** Rosana E. De Castro, Ansgar Poetsch

**Affiliations:** ^1^Instituto de Investigaciones Biológicas (IIB-CONICET-UNMDP), Universidad Nacional de Mar del Plata, Mar del Plata, Argentina; ^2^College of Marine Life Sciences, Ocean University of China, Qingdao, China; ^3^Queen Mary School, Medical College, Nanchang University, Nanchang, China; ^4^Plant Biochemistry, Ruhr University Bochum, Bochum, Germany

**Keywords:** cell envelope proteases, membrane proteases, proteolysis, prokaryotes, bacteria, archaea

Proteolysis plays a fundamental role in cell physiology and is exerted by enzymes (proteases or peptidases) that cleave or degrade protein or peptide substrates. Proteases localized in the context on the prokaryotic cell envelope are implicated in cell signaling, regulation of gene expression, protein quality control, protein export and membrane anchoring, biogenesis/remodeling of the cell wall and surface structures, antibiotic resistance mechanisms among other key cellular processes. These membrane proteases belong to various protease families, show different catalytic mechanism, and can have their active site at/outside the membrane surface in an aqueous environment (cytoplasm, periplasm, extracellular medium) or embedded within the lipid bilayer (Wolfe, 2009; Dalbey et al., 2012). The first group includes signal peptidases (SP), site-1-proteases (S1P), HtpX and AAA+ proteases FtsH and the archaeal type-LonB. The second group, denoted as Intramembrane Cleaving Proteases (ICliPs or IMPs) is represented by the rhomboid family, site-2 proteases (S2P), GxGD-aspartyl proteases (presenilin and eukaryal signal peptide peptidase SPP), and Rce1- type glutamyl proteases (Sun et al., 2016).

The goal of this Research Topic was to advance knowledge and stimulate/promote discussion on different aspects of the biology of the proteolytic systems that occur in the context of the cell envelope of prokaryotes.

Compared to other proteases, there is a much bigger gap in research for those that occur in the prokaryotic cell envelope. First, while many of the membrane protease families are conserved in the three domains of life (e.g., the Rhomboid family) they have been studied mainly in eukaryotes and to a more limited extent in prokaryotes. Second, our current knowledge on the archaeal protease biology (function, targets) is comparatively much limited. Obvious open questions are how these proteases recognize substrates and whether this involves mechanisms/motifs unique to prokaryotes. Concerning protease function and targets, there is a dire need for more studies covering a broad range of prokaryotes to adequately inform about range as well as conservation of targets and functions, in particular those important for biomedicine and biotechnology.

The original research articles and mini-review in this Issue describe work that address current research gap by extending our understanding of the substrate recognition mechanisms, diversity, and biological function of proteases localized in the cell envelope of various prokaryotic organism. These articles exemplify how modern analytical tools such as proteomics have facilitated the identification of potential targets and contribute to unraveling the biological function of membrane proteases in bacteria and in some archaea.

Site-2- Proteases (S2P) participate in Regulated Intramembrane Proteolysis (RIP) in various organisms. In *E. coli*, S2P protease RseP regulates an extracytoplasmic stress response through a mechanism that involves the sequential cleavage of the membrane-spanning anti-σ factor (anti-σ^E^) RseA first by the S1P protease DegS, which cleaves RseA periplasmic domain, and then by RseP, allowing the release of σ^E^ and subsequent gene activation. The periplasmic PDZ domains of RseP act as a filter to exclude the intact substrate RseA from the active site of RseP. In his work, Miyake et al. provide insights on the substrate recognition mechanism of RseP protease. They showed that an amphiphilic segment of RseP downstream the PDZ domains and located in the periplasmic surface of the membrane (helix H1), directly interacts with the DegP-cleaved form of RseA, facilitating its discrimination by the PDZ-domains of RseP. The authors propose that H1 is important for the proteolytic function of RseP as it relates to the PDZ-mediated discrimination of its substrate and contributes to its proper positioning and cleavage.

The cell wall is critical for bacterial survival. Its main component is the peptidoglycan, a highly crossed-linked heteropolymer of glycans and short peptide chains. This structure is modified/remodeled by the action of peptidoglycan hydrolases, which are very diverse in specificity and structure. Autolysins are implicated in cell wall metabolism during bacterial growth, division and elongation while bacteriocins act in the elimination of related species living in the same niche. Using biochemical and genetic approaches, Wysocka et al. characterized two novel and distinct peptidoglycan hydrolases belonging to the M23 family of metallopeptidases derived from *Staphylococcus pettenkoferi*. While these enzymes shared some traits, including significant sequence identity, structural organization and conservation of catalytic residues, they showed distinct gene distribution and isoelectric point. The potential biological function of these novel enzymes was discussed.

The Rhomboid family of IMPs are conserved across the three domains of life, however, their biological function and natural substrates are scarcely known in prokaryotes. To expand knowledge on this issue, Luenenschloss et al. obtained strains mutated in two rhomboid genes in the bacterium *Corynebacterium glutamicum* and characterized these strains phenotypically and examined their proteome, transcriptome and lipidome. They observed abundance changes in enzymes involved lipid biosynthesis (mycolic acids and others) and well as in lipid composition, providing evidence of a potential role of rhomboids in modifying cell wall composition in this organism.

Archaea are a phylogenetically distinct and physiologically diverse group of prokaryotes. They are widely distributed in nature but are unique in its ability to survive in the most extreme environments. Most of the membrane protease families that occur in eukaryotes and bacteria are conserved in archaeal genomes, however, some of these proteases remain uncharacterized and/or their physiological role and targets remain to be discovered. The minireview by De Castro et al. summarizes advances on membrane localized-proteases in the model archaeon *Haloferax volcanii*. Combining genetic, physiology and/or proteomics approaches potential targets and cellular processes affected by the membrane-anchored LonB and Rhomboid proteases were identified. LonB appears as a key regulator of carotenogenesis while both proteases are likely implicated in protein glycosylation. Advances on the S-layer glycoprotein maturation mechanism mediated by archaeosortase ArtA are discussed.

Research on different aspects of membrane proteases in all kinds of organisms, including prokaryotes, will contribute to a deeper understanding of the catalytic mechanisms and biological functions of these proteolytic systems as well as promote the design of strategies to control the development of disease and expand resources for Biotechnology.

## Author contributions

RD and AP have both contributed to the Editorial and approved the submitted version.

## Conflict of interest

The authors declare that the research was conducted in the absence of any commercial or financial relationships that could be construed as a potential conflict of interest.

## Publisher's note

All claims expressed in this article are solely those of the authors and do not necessarily represent those of their affiliated organizations, or those of the publisher, the editors and the reviewers. Any product that may be evaluated in this article, or claim that may be made by its manufacturer, is not guaranteed or endorsed by the publisher.
